# Point-of-Care Diagnostic Services as an Integral Part of Health Services during the Novel Coronavirus 2019 Era

**DOI:** 10.3390/diagnostics10070449

**Published:** 2020-07-03

**Authors:** Tivani P. Mashamba-Thompson, Paul K. Drain

**Affiliations:** 1 Department of Public Health, University of Limpopo, Polokwane, Limpopo Province 0727, South Africa; 2 International Clinical Research Center, Department of Global Health, University of Washington, Seattle, WA 98195-7965, USA; pkdrain@uw.edu; 3 Division of Infectious Diseases, Department of Medicine, University of Washington, Seattle, WA 98195-7965, USA; 4 Department of Epidemiology, University of Washington, Seattle, WA 98195-7965, USA; 5 Department of Surgery, Harvard University, Massachusetts General Hospital, Boston, MA 02114, USA

**Keywords:** point-of-care diagnostics, healthcare services, COVID-19 era

## Abstract

Point-of-care (POC) diagnostic services are commonly associated with pathology laboratory services. This issue presents a holistic approach to POC diagnostics services from a variety of disciplines including pathology, radiological and information technology as well as mobile technology and artificial intelligence. This highlights the need for transdisciplinary collaboration to ensure the efficient development and implementation of point-of-care diagnostics. The advent of the novel coronavirus 2019 (COVID-19) pandemic has prompted rapid advances in the development of new POC diagnostics. Global private and public sector agencies have significantly increased their investment in the development of POC diagnostics. There is no longer a question about the availability and accessibility of POC diagnostics. The question is “how can POC diagnostic services be integrated into health services in way that is useful and acceptable in the COVID-19 era?”.

Point-of-care (POC) refers to the location where healthcare interventions are carried out. These interventions can be carried out in a variety of settings including in the home, in the office, in the community and at a healthcare facility. Disease diagnosis or testing is one of the healthcare interventions that can be carried at POC and referred to as POC diagnostic services or POC testing. POC testing is performed using various POC diagnostics to enable the near-patient detection and monitoring of disease conditions in order to inform prognoses, guide treatment choices and predict treatment responses [1]. The advent of POC diagnostics in resource-limited settings has enhanced diagnostic capacity and helped to improve access to healthcare in areas where disease burden is high and diagnosis remains a weak point in the healthcare system [2,3,4]. The most commonly used and accessible POC diagnostics in most of these settings are pathology tests such as HIV and malaria tests [5,6]. This issue has demonstrated the use of pathology, radiological and information technology systems as well as mobile technology and artificial intelligence for POC diagnostic services. The advent of the novel coronavirus 2019 (COVID-19) pandemic has put POC diagnostics in the spotlight and prompted rapid advances in the development of POC diagnostics and their delivery approaches. Global private and public sector agencies have significantly increased their investment in the development of POC diagnostics. There is no longer a question about the availability, accessibility and acceptability of point-of-care diagnostics services. The question is “how can point-of-care diagnostics services be integrated into current health services in way that is useful and acceptable in the COVID-19 era?”. This issue presents translational research presenting evidence of the benefits of implementing POC diagnostics and strategies to help integrate POC diagnostic services into current healthcare services.

The development of new evidence-based POC diagnostics and the replication of these diagnostics to extend their reach is a global health priority. The appropriate integration of new POC diagnostics approaches is crucial for ensuring desirable outcomes. The implementation of POC healthcare interventions such as POC testing ought to be relevant to each specific context and sensitive to local culture. Factors such as infrastructure, resources, values and the characteristics of the participants can influence the implementation, scalability and sustainability of health interventions. A study conducted in Nigeria calls for community education, screening for schistosomiasis, and the enhancement of diagnostic capacity and strengthening of the capability of health workers through point-of-care diagnostics [2]. Our previous research demonstrates the need for improving the accessibility of Glucose-6-Phosphate Dioxygenase Deficiency [7] and blood-group and rhesus-type tests [8] as part of antenatal care services in malaria regions. These studies also highlight the need to update the World Health Organization (WHO) essential diagnostics (EDL) and for the development of content specific for POC diagnostics lists during the COVID-19 era. Following the implementation of recommended diagnostics at the POC, there is a need for optimizing the development of POC diagnostics delivery approaches to ensure continual quality service delivery, particularly among underserved populations and resource-limited settings.

Research suggests the need for training healthcare workers to improve POC diagnostic service delivery in resource-limited settings [9,10,11]. Primary Healthcare (PHC) healthcare workers in rural South Africa suggested an experiential learning approach using eLearning to help them maintain their competence in terms of HIV POC diagnostics service delivery [12]. Despite the wide availability of PHC-based HIV testing services, there are still substantial gaps. Access to these services is a challenge to key populations such as men in sub-Saharan Africa. A study conducted in Rwanda focusing on optimizing the implementation and scale up of HIV self-testing approaches for HIV to help improve men’s engagement with HIV services has identified the following priority areas: the creation of awareness; the training those involved in the implementation process; the regulation of the selling of the self-test kits; the reduction of the costs of acquiring the self-test kits through the provision of subsidies; and ensuring the consistent availability of the self-test kits were identified [13]. Previous research shows that the advancement of mobile technology and improved data affordability has benefited the successful implementation of POC diagnostics approaches such as self-testing [14]. Smartphone technology and POC ultrasound (POCUS) devices have proven to be key examples of how technological advances are poised to improve healthcare delivery in resource-limited settings [11,15]. POC diagnostics has the potential help with the much-needed rapid development of information systems within the health sector through artificial intelligence and machine learning-linked POC diagnostics [16]. The integration of POC diagnostics with existing Health Information Systems should help to improve disease diagnostics and management [17].

The integration of available POC diagnostics into our current healthcare service needs to be prioritized to aid the prevention and management of current pandemics and in preparation for future pandemics. It is clear that the successful implementation of point-of-care diagnostics requires a transdisciplinary approach. Investment in transdisciplinary research platforms for POC diagnostics is recommended. These platforms can also foster improved awareness and recognition of POC diagnostics services as a standalone healthcare service and development of POC diagnostics curricula for the training of a new cadre of healthcare workers, dedicated to POC diagnostic services. The successful implementation of such platforms requires multidisciplinary and multi-sectorial stakeholder involvement including higher education institutions, diagnostics and information and mobile technology developers and providers as well as implementers and users of POC diagnostic services.
